# Characteristics of Children Who Lost the Diagnosis of Autism: A Sample from Istanbul, Turkey

**DOI:** 10.1155/2014/472120

**Published:** 2014-04-27

**Authors:** Nahit Motavalli Mukaddes, Mustafa Deniz Tutkunkardas, Oktay Sari, Aydan Aydin, Pınar Kozanoglu

**Affiliations:** ^1^Child Psychiatry Department, Istanbul School of Medicine, Istanbul University, 34080 Istanbul, Turkey; ^2^Istanbul Institute of Child & Adolescent Psychiatry, 34365 Istanbul, Turkey; ^3^Department of Special Education, Marmara University, 34730 Istanbul, Turkey; ^4^Private Education Center, 34140 Istanbul, Turkey

## Abstract

*Aim.* The aim of this study was to describe a group of children who lost a diagnosis of autism following participation in early educational programs. *Method.* This is a descriptive study reporting the characteristics of children (*n*: 39) who lost their diagnosis of autism and explaining the educational programs that these children followed. The data were collected by reviewing the participants' files and through examinations. *Results.* All of the children were placed at regular psychiatric follow-ups. The mean age at referral was 2.39±0.75 years, whereas the mean age at the time of optimal outcome reported was 5.11 ± 1.95 years. Two of the children were in early intensive behavioral intervention (EIBI), and the rest were in a comprehensive naturalistic behavioral program. The childhood autism rating scale (CARS) total scores at baseline and final were 32.75 ± 3.15 and 18.01 ± 1.76, respectively. The mean IQ of the group at final examination was 116.70 ± 18.88. *Conclusion.* It could be concluded that a group of children with an autism diagnosis could lose the diagnosis of autism upon early intervention. High IQ and the development of communicative and language skills at an early age could be the most powerful factors contributing to an optimal outcome.

## 1. Introduction

Autism spectrum disorders (ASD) are a group of disorders characterized by impairments in reciprocal social interaction, communication, restricted interests, and stereotypical behaviors [1].

Several studies on the consequences of ASD demonstrated unfavorable outcomes for the majority of individuals with a diagnosis of ASD. In an early longitudinal study, it was reported that 60% of subjects with ASD have a poor outcome and are severely handicapped, 35% have a “fair or good” adjustment, and only 1.5% were functioning normally [2]. Recent long-term studies on outcomes demonstrate unfavorable results for low-functioning individuals with autistic disorder (AD) [3, 4] and a favorable outcome in the majority of individuals with high-functioning autism (HFA) and Asperger's disorder (AS) [4, 5].

Over the last few decades, there has been an increase in the number of publications on the efficacy of different intervention programs. Improvements in the core symptoms of ASD, cognitive abilities, and adaptive functions have been reported in these studies [6–13]. Although these studies reported improvements in many aspects of autism, there are a limited number of studies that report “recovery” or optimal outcomes.

The first study in this field was performed by Lovaas [6], in which 47% of children with a diagnosis of autism who were in Applied Behavior Analysis (ABA) achieved the average IQ and could be placed in a regular educational situation without any specialized support [6]. Lovaas used the term “recovery” or “best outcome” to describe these children. Following this study, other investigations assessing the effectiveness of early behavioral approaches indicated that a substantial minority of children (ranging from 27% to 48%) perform within the average range in posttreatment assessments [14].

However, Mundy criticized Lovaas' approach, concluding that having average (or higher) IQ and attendance to a regular school program were not sufficient for “recovery.” Many high-functioning children with a diagnosis of autism spectrum disorders may also possess an average or high IQ and be able to attend regular classrooms [15].

Among studies that assessed the effectiveness of different interventional approaches for autism's core symptoms, only some reported a “loss of diagnosis” or “optimal outcome.” One of these studies was performed by Sallows and Graupner [8]. They compared the effectiveness of clinical and parent-directed behavioral intervention. After four years of treatment; they assessed outcomes, including cognitive, language, adaptive, social, and academic measures that were similar among groups. After combining groups, they reported that 48% (11 of 23) of all children showed “rapid learning.” “Rapid learners” scored normally on IQ, language, adaptive functioning, and school placement tests. Additionally, 8 of the participants did not meet the criteria for an ASD according to the ADI-R [8]. According to Helt et al., these 8 individuals met the criteria for an optimal outcome [16]. The other important study in this field was performed by Fein et al. [17]. They reported eleven children with an earlier diagnosis of ASD who lost the diagnosis of ASD and developed attention deficit hyperactivity disorder (ADHD). Eight had received ABA treatment, and 3 had received intensive preschool classroom intervention. They reported that this group was at risk for significant attention problems as well as some subtle social difficulties and perseverative interests. Recently, Fein et al. published another comprehensive study assessing the functioning of 34 individuals with a previous history of autism. They compared the functioning of the optimal outcome (OO) group with individuals with high-functioning autism and a typical developing (TD) group. They reported that the OO and TD groups' mean scores did not differ regarding socialization, communication, face recognition, or most language subscales. They concluded that their result substantiates the possibility of an optimal outcome from ASD and demonstrates an overall level of functioning within normal limits for this group [18].

Another article in this field is a retrospective study reporting “recovery” from autism in thirty-eight individuals who received early intensive behavioral intervention [19].

In addition to studies that reported a loss of diagnosis of autism after intensive behavioral treatment, Zappella [20, 21] reported “reversible autism” in a group that had never been in behavioral treatment. First, he described a case series, all young males who showed autistic regression after age one. They all had motor and vocal tics. Autistic symptoms disappeared after naturalistic approaches and guidance; however, the children developed Tourette disorder [20]. In his later paper, Zappella described a heterogeneous group of children with a former diagnosis of pervasive developmental disorders (PDD) who lost their PDD diagnosis. Part of this group had concurrent tics and/or ADHD. Some had electroencephalographic (EEG) abnormalities comparable with Landau-Kleffner Syndrome (LKS). He stated that 7.3% of this group recovered spontaneously. It was also mentioned that none of the recovered individuals received ABA and that all underwent a more naturalistic treatment program [21].

Despite an increase in studies demonstrating an optimal outcome in autism, many professionals in this field have a skeptical approach to the idea of “loss of diagnosis” in ASD. Some believe that individuals with OO are initially misdiagnosed. However, evidence regarding similarities in the baseline between the optimal outcome group and persistent ASD in terms of social, communicative, and cognitive functions [22], as well as in head circumference as a biomarker of autism [23], supports the hypothesis of optimal outcome in autism. A recent comprehensive study reported no difference between an optimal outcome group and a typically developing group in socialization, communication, face recognition, or most language subscales; this clearly demonstrated the possibility of optimal outcomes in individuals with a history of autism [18].

As current evidence elucidates, there are only a limited number of studies that report and describe the characteristics of children who have lost a diagnosis of ASD. While some of these studies report optimal outcomes in groups that received intensive early intervention, only one study reported spontaneous recovery.

Therefore, the aim of this study was to describe the characteristics of subjects who lost the diagnosis of autism and to explain the interventional programs that they followed.

## 2. Methods and Material 

This is a descriptive study to describe the characteristics of individuals who were previously diagnosed with ASD and who lost their diagnosis and achieved an optimal outcome.

### 2.1. Definition of “Recovery”/Optimal Outcome

To the best of our knowledge, the criteria that Helt et al. [16] defined are the only criteria that include either the loss of core symptoms of autism or the demonstration of an IQ in the normal range and normal adaptive functioning. Because our aim was to define a homogenous group that achieved a normal range of developmental trajectory in cognitive, social, and communicative areas, we did not include the group that lost the diagnosis of autism but met the criteria for language disorder and/or intellectual disability.

Our definition of optimal outcome was similar to that of Helt et al. [16]. We considered a case to have achieved an optimal outcome if he/she met the following criteria at baseline: (1) having a diagnosis of ASD at baseline based on DSM-IV criteria, (2) the presence of language delay (no words by 18 months and no word combinations by 24 months), and (3) total Childhood Autism Rating Scale (CARS) [24, 25] score over 25.5 based on scores reported by Chlebowski et al. [26], as well as these criteria at final assessment: (1) not meeting the criteria for any type of ASD based on DSM-IV, (2) not needing special education for the core symptoms of autism, (3) total score of CARS and Autism Behavior Checklist (ABC) [27, 28] in the nonautistic range, and (4) IQ in the nonretarded range (over 78).

### 2.2. Participants

Participants in our study were the regular patients of a psychiatry clinic, which is a referral center for developmental neuropsychiatric disorders. We included children who previously received a diagnosis of ASD and who did not meet the criteria for any ASD in the final examination. There were thirty-nine individuals (30 males and 9 females) with an age range of 18–54 months at referral. Thirty-eight met the full criteria for AD, and one met the criteria for pervasive developmental disorder-not otherwise specified (PDD-NOS) diagnosis at baseline according to DSM-IV-TR. Their age range at the time of final assessment was 3–10 years old.

### 2.3. Diagnosis and Follow-Up Procedure

The first assessment protocol was applied to all referrals with a probable diagnosis of ASD. The first assessment included an in-depth psychiatric examination of the child by taking detailed information from parents, watching home videos, and observation of the child's interaction with parents in a playroom. A diagnosis of ASD was made, based on DSM-IV-TR criteria, and the severity of autistic symptoms was measured using CARS. The Ankara Developmental Screening Inventory [29] was used to assess the social, self-help, communicative abilities and gross and fine motor skills of the child. A medical examination, including an auditory test, metabolic screening, and fragile X syndrome screening, was conducted.

The first diagnostic assessment of patients revealed that 39 individuals met the full criteria for AD and that 2 met the full criteria for PDD-NOS according to the DSM-IV-TR. Their CARS scores were between 26.5 and 43. The data of 2 children who had medical problems (auditory impairment and hypothyroidism) were excluded from the study. Therefore, we included 38 individuals with an initial diagnosis of autism and 1 with a diagnosis of PDD-NOS.

After receiving an ASD diagnosis, all children were referred to an education program.

Children were followed up psychiatrically every 3-4 months. In each follow-up visit, the examiner evaluated the child's social-communicative skills and behavioral patterns by watching home videos, observing the child in office, collecting information from parents, and reading written reports from senior educators. Each assessment aimed to evaluate ASD symptoms, such as social-communicative impairment, the severity and frequency of repetitive behaviors, self-help skills, and adaptive behaviors, as well as additional behavioral and medical problems. In addition to the progression of the child, the quality of the education program was also assessed based on information from parents' and educators' written reports. After every examination, a child psychiatrist shared their feedback with the senior educators and supervisors of the education program. Additionally, psychiatric follow-up helped parents to overcome their anxieties and concerns about their child's problems. During follow-up, when a child did not meet the criteria for any type of ASD according to the DSM-IV-TR, outcome measures were applied.

### 2.4. Measurements

(1) The Childhood Autism Rating Scale (CARS) is a behavioral rating scale used to assess the presence and severity of autism spectrum disorders [24]. It has been shown to have good sensitivity and specificity to distinguish ASD from non-ASD and other developmental disorders in children as young as 2 years of age [26]. A reliability and validity study of CARS was performed in Turkish [25]. CARS consists of 15 items that assess the severity of social-communicative impairments; therefore, CARS could be used for assessing and grading reductions in social-communicative impairments.

(2) The Autism Behavior Checklist (ABC) [27] consists of 57 behaviors that appear to be more commonly observed in autistic children than in children with other handicaps. The ABC was translated into Turkish, and reliability and validities studies were performed [28]. The total scores generated using the ABC ranged from 0 to 158. A total score of 67 or above was considered to indicate autism with “high probability.” Parents were asked to fill out this scale during the final phase.

(3) The Ankara Developmental Screening Inventory (ADSI) [29] is a 154-item scale widely used in Turkey for the assessment of social, cognitive, and communicative abilities. The ADSI includes four subscales: the language-cognitive subscale, the fine motor subscale, the gross motor subscale, and the social interaction and self-care abilities subscales. In this study, educators utilized the scale during certain intervals (every 4–6 months) to evaluate the child's progression in these areas and to determine and tailor their educational program. Because it was not administered at a uniform point in time, it is not possible to report the result of the ADSI.

(4) IQ tests (the Stanford-Binet Intelligence Scale [30] or the Wechsler Intelligence Scale for Children-Revised (WISC-R) [31, 32]) were used to assess cognitive abilities.

### 2.5. Educational Program

The government of Turkey only funds 2 hours of the educational program for ASD per week in Turkey. The rehabilitation centers that provide this limited educational service usually do not have sufficient well-trained professionals who are familiar with ABA or other more effective behavioral treatments. There are a few institutes/schools that employ early intensive behavioral intervention (EIBI). The Turkish government does not provide funding for these programs, and a 20-hour per week program increases the financial burden on the family by €3,000 to €5,000 per month. There are also a few private centers that provide different modalities of educational programs (floor time, individualized social skills training, etc.) for children with ASD.

The patients in our clinic were primarily from upper-middle income families; thus, they had ample access to educational resources. Nevertheless, only 2 patients in this group were able to attend ABA programs with frequencies ranging from as low as 8 hours per week up to the recommended 20 hours per week.

Thirty-seven individuals attended a comprehensive educational program, which is inspired by Pivotal Response Training (PRT) [33]. This program executes the principals of behavioral treatment and acquired abilities with a special focus on social-communicative development. It requires a more active collaboration with parents and other available family members. The program aims to enrich the environment and promote social responsiveness by working on eye contact, joint attention, imitative abilities, and pretend and make-believe plays. Other important goals include the prevention of interfering behaviors, practicing undeveloped skills, and the enhancement of verbal abilities.

Due to the scarcity of well-trained educators in this field, the senior educators who led the program could only see each patient for 1–3 sessions per week; however, these educators provided parents with intensive home programs and asked them to work with their children as much as they could. If parents struggled with the application of the home program, trained students were sent to their home to work with the child and to supervise the parents' interaction. These students were final-year students of special education programs. They had all observed their senior educators work with children with ASD for at least 6 months and had weekly supervision meetings with their senior educators.

As stated previously, this program required all available family members to be engaged; thus, they were encouraged to take responsibility for working with their child. We also encouraged healthy siblings and cousins to take part in the education program, at least as a “play partner.”

Children who showed increased social responsiveness in individual education sessions and who showed progression in maintaining eye contact, joint attention, and imitative abilities were sent to the nursery to practice these abilities in a natural environment. The interval between intake and transfer to the nursery varied greatly between the children and ranged from 4 months to 19 months.

Although this education program works on enhancing verbal abilities, if a child failed to develop meaningful words by the age of 26–30 months, speech therapy was initiated.

In short, this is an individualized, comprehensive program that is widely applied across different settings and is in some respects similar to PRT.

**Figure 1:**
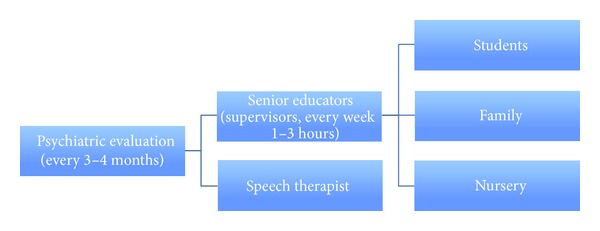
Providers of the educational program.

A child psychiatrist who has 20 years of research and clinical background with ASD conducted the clinical diagnoses and follow-ups. A blind examiner, who is a child psychiatrist with 5 years of experience in the autism field, reviewed the records of all children at baseline and at the time of optimal outcome. Only individuals about whom both psychiatrists agreed regarding baseline diagnosis and optimal outcome were included in the study. The senior educators were educators with Ph.D. and M.S. degrees in special education and who had at least 10 years of experience in working with young children with ASD. These senior educators supervised the home program and the nursery program. They led this program in continuous consultation with child psychiatrists. See Figure 1 for the review of the providers of the educational program.

Because the behavioral interventions were not standardized or uniform, no treatment fidelity measures could be taken. However, parents were regularly consulted, and interventional progressions were periodically checked (every 4–6 months) using ADSI.

### 2.6. Statistical Analysis

All data were organized in Microsoft Excel 2010, and all statistical tests, excluding the correlation analysis, were conducted in Excel. Differences between means were calculated using a *t*-test. A *P* value <0.05 was regarded as statistically significant. For multiple comparisons for changes in each CARS item, a Bonferroni correction was conducted, and a *P* value < 0.0033 was considered statistically significant. Relationships between parametric values were assessed using the Pearson correlation coefficient in the Statistical Package for Social Sciences (SPSS) 16.0, SPSS Inc., Chicago, Illinois, USA.

## 3. Results

The mean age at referral was 2.39 ± 0.75 years (range: 1.5–4.5 years), which was also the age to start special education. The children started to talk with meaningful words at the age of 2.46 ± 0.92 years (range: 1.5–4.5 years) and used phrases at the age of 3.20 ± 0.91 years (range: 2–5 years). The mean age at optimal outcome was 5.11 ± 1.95 years. The mean number of psychiatric interviews from intake to the time of optimal outcome was 8.47 ± 3.89 sessions. Some patients are being followed up for other psychiatric problems after loss of ASD diagnosis. The education program of patients is summarized in Table 1.

The initial mean CARS score was 32.75 ± 3.15, and the CARS scores in the final evaluation decreased to 18.01 ± 1.76 (*P* = 0.000). The difference between the initial and final CARS scores was not related to the number of special education sessions per week, age at the start special education, or the final IQ. See Table 2 for the changes in CARS items.

The final mean IQ score of the patients was 116.70 ± 18.88 (range: 80–148), and the final ABC score was 7.53 ± 7.83. The results of the outcome measures are summarized in Table 3.

The time from baseline to optimal outcome was 2.71 ± 1.67 years (range: 0.5–8 years).

Because some patients lost their diagnosis relatively quickly (8 patients required ≤1 year of intervention), a possible relationship between the time required to achieve optimal outcome and the number of weekly special education sessions, age at diagnosis, initial CARS score, IQ, and age of development of meaningful words and communicative phrases was examined. A negative relationship between the final IQ and the length of time between the initial diagnosis and optimal outcome was observed. That is, the higher the final IQ, the less the time required for achieving an optimal outcome. Additionally, a positive relationship was found between the age of language development and time to optimal outcome. There was no statistically significant relationship between the length of time to optimal outcome and the age at intake, CARS initial total score, or the number of weekly sessions. See Table 4 for details.

Of the 39 patients, 23 (58.9%) had an additional psychiatric disorder: 46.1% (18 patients) had ADHD, 23.07% (9 patients) had some type of anxiety disorder, and 7.69% (3 patients) had some type of tic disorder. Twenty-three patients (58.9%) were on medications with an average of 1.69 ± 0.82 medications per patient. Psychiatric comorbidity was assessed in psychiatric follow-up (every 3-4 months). The results presented here demonstrate the psychiatric comorbidities from initial intake until optimal outcome.

## 4. Discussion

This study documents the characteristics of children and the intervention programs that achieved an optimal outcome. The description and identification of factors that led to a favorable outcome in this group is a very important issue because it may shed light on the neurobiology of autism, provide information regarding the effectiveness of intervention programs, and also illuminate potential characteristics of children who may achieve an optimal outcome.

However, prior to discussing the characteristics of this group, it may be more pertinent to discuss diagnostic issues.

The first question that needs to be answered is whether the OO group was initially misdiagnosed. The early age at referral and therefore diagnosis at early age naturally raises a question regarding the probability of misdiagnosis. Psychiatric assessments of this group were performed in detail and were based on home videos, observations of child-parent interactions in play situations, and detailed developmental histories from parents. Additionally, we used instruments, such as CARS, that can distinguish between children with ASD and those with non-ASD. The psychiatric follow-up and assessment were repeated every 3-4 months. All psychiatric assessments and follow-ups were performed by a child psychiatrist with 20 years of experience in the field of autism. It is now agreed that clinical judgment by an experienced clinician is considered to be the “gold standard” for the diagnosis of autism in young children. Therefore, the probability of misdiagnosis is not likely.

The second question in this area is related to the diagnostic stability. The young age at referral also raises a question about the diagnostic stability. Some studies showed high rates of diagnostic stability (85%–89%) when autism was diagnosed at the age of 2 [34–36]; however, a study later showed lower diagnostic stability (63%) when autism was diagnosed at the age of 2 [37]. Another study reported that 18% of children with a diagnosis of ASD at age 2 lose the diagnosis of ASD by age 4. The group in that study was in an intervention program [23]. Our group was also in an intervention program and, despite the program, only 9 individuals moved off the autism spectrum prior to age 4, while 30 moved off after age 4. Taken together, it could be said that maturation may have contributed to improvements in social-communicative and cognitive areas [21]; however, we are not certain if the children in our group and other studies would have moved off the spectrum without any intervention. The important issue that we should consider here is the cohort effect. Increased awareness about autism among parents leads to patient referral at a younger age, with milder problems in social-communicative areas. It should be noted that children who are referred at an early age with major problems in one area of development often show deficits in several domains. Therefore, there is also the possibility of a different clinical manifestation later in life that may explain the developmental trajectory of some children who initially present with ASD.

The third point that needs to be clarified is the role of the inherent characteristics of the children, families, and intervention programs of this group. Although the mechanisms of improvement for any given child are not known, it could be assumed that the interaction of the child's own characteristics with an appropriate intervention program contributes to this favorable outcome.

We will discuss the inherent characteristics of children, families, and treatments in this group.

### 4.1. Age at Referral

Patients in this group were referred to us at an early age (mean age: 2.35 ± 0.75); 30 of 39 (76%) were referred at the age of 18–30 months. We assumed that the young age at intake could be one of the factors contributing to favorable outcome. Surprisingly, we did not find a statistically significant correlation between the age of intake and the length of time to achieve OO. Therefore, this study failed to show that an earlier age at intake leads to an earlier loss of diagnosis. Harris and Handleman [38] examined the predictive power of age and IQ at the time of admission to intensive behavioral treatment. They reported that a younger age and a higher IQ were predictive of placement in a regular education class [38]. Turner [35] examined the age 2 predictor of outcome at age 9 and reported that 70% of the children diagnosed prior to the age of 30 months had “more optimal” outcomes, while only 28% of children diagnosed older than 30 months achieved “more optimal” outcomes. The lack of a control group in our study limits our interpretation. Despite some controversy regarding the effect of early identification on outcome, it is accepted that an intervention, which is sufficiently early to coincide with the maximum neural plasticity, contributes to residual normality in various neurodevelopmental disorders [39].

### 4.2. Symptom Severity

Reviewing the scores of CARS items indicates the presence of mild to moderate impairment in all items at baseline. One important point is the statistically significant change from baseline to the time of optimal outcome in all autism-specific items. Thus, this group showed a great reduction in the core symptoms of autism from baseline to the time of optimal outcome. There were no statistically significant changes in only 2 items, which are not among the core symptoms of autism, namely, item number 10 (fear or nervousness) and number 13 (activity level).

According to the CARS total score, thirty-five of our patients (90%) were rated as having mild to moderate autism, and only 4 were rated as having severe autism. Therefore, it appears that mild to moderate autism was a common characteristic of the majority of our optimal outcome group. Our result is comparable with the results of Turner in which they showed that children who lost the diagnosis of autism at age 4 had a lower total CARS score at age 2 than a group that showed diagnostic stability [37]. Unfortunately, the lack of a control group in our study limits our interpretation of the effect of symptom severity on outcome. We examined the relationship between initial CARS scores and the length of time to optimal outcome. However, we did not observe a statistically significant relationship.

The influence of symptom severity on outcome is a controversial subject. While some studies claim that there are no significant effects of symptom severity on outcome [22], others mention that children with “milder social impairment” are more likely to show favorable outcomes [37]. In a recent study by Fein et al., the optimal outcome group had “milder autism” in childhood and “the milder presentation applied to social area but not communication and repetitive behaviors areas” [18]. Taken altogether, it could be suggested that individuals with milder symptoms are more likely to achieve optimal outcomes.

### 4.3. Early Communication and Language Development

Children in our group began to speak with meaningful words at the age of 2.47 ± 0.81 years and used phrases at the age of 3.15 ± 0.85 years. It was found that the time until optimal outcome was positively related to the first use of words (Pearson correlation coefficient: 0.607, *P* = 0.027) and first phrases (Pearson correlation coefficient: 0.734, *P* = 0.006). In other words, children who started to speak at a younger age more quickly achieved an optimal outcome. Improvements in verbal and communicative abilities are known as the most consistent prognostic factors [40]; thus, the age-appropriate acquisition of spoken language skills is an important factor leading to a favorable outcome. Naturally, children with verbal and communicative abilities have a greater chance to interact with other people—including peers—than nonverbal children. The presence of verbal abilities inevitably enhances social interaction and ultimately has a positive influence on outcome.

### 4.4. Intelligence Quotient (IQ)

Although we could not test the initial IQ of the entire group, their final IQ scores point to their high intellectual potential. A total of 62.9% of the group had an IQ over 110, and the mean IQ of the group was 116.70 ± 18.8. Additionally, the relationship between IQ and the length of time from intake to loss of diagnosis showed a negative correlation. In other words, children with higher IQ scores achieved optimal outcomes more quickly than other children. This is consistent with previous studies that reported IQ as the strongest prognostic factor for outcomes of children with ASD [17, 18] due to expressive and receptive language skills [40]. Finally, other studies on optimal outcomes reported higher than average IQ in their OO group [17, 18]. Taking all factors into consideration, the hypothesis that higher IQ scores facilitate recovery appears to be plausible.

### 4.5. Absence of Medical Disorders

It is known that disorders affecting the central nervous system, such as epilepsy [41], have a negative influence on outcomes [42]. The absence of such disorders in this group appears to be another factor that influences outcomes.

### 4.6. Role of Intervention Program

The educational programs in which our patients participated were home-based educational interventions. Although we aimed to provide an intensive home program, our information about the intensity of the home program is limited to what parents reported to us (except for families that had home teachers). For that reason, we only considered the number of weekly in-office sessions that the patients had with education program supervisors.

The lack of a control group limits our interpretation of the effectiveness of the intervention program. Furthermore, assessing the relationship between the number of weekly sessions with educators and the length of time to optimal outcome failed to show a statistically significant difference. Finally, there was no statistically significant relationship between the number of special education sessions per week and the change in CARS score.

Previous studies reported OO in children who attended an ABA program [17, 19]. To the best of our knowledge, one study also reported recovery in which patients received only naturalistic, relation-based interventions [21]. In our group, only 2 individuals attended the ABA program, and the rest received home-based naturalistic behavioral interventions.

Our results may demonstrate the utility of developmentally appropriate, naturalistic behavioral programs in some toddlers with a diagnosis of ASD. However, intervention alone did not independently lead to a positive outcome. While intervention is highly important, it should be noted that the prognosis depends on the relationship between the child's characteristics and the intervention program.

### 4.7. Role of the Families

The parents of these children played an important role in each child's education. According to the clinical impressions of both the psychiatrists and educators, all parents in this group showed high motivation and great effort. However, it is important to remember that the majority of these families were comprised of well-educated parents from the upper-middle class. They showed excellent collaboration with all team members. In 9 of these families (23.07%), the loss of the diagnosis of ASD was relatively rapid (prior to the age of 4). However, the remaining families (30 patients, 76.9%) had to work diligently until their children were older (between the ages of 4 and 10).

One important point that affects the generalizability of the results of this study pertains to family characteristics. Because the majority of these parents were well-educated individuals with sufficient economic means, they provided a high quality assessment and intervention program for their children; they proficiently monitored and organized the program and had a very close collaboration with both the psychiatrist and educators.

In summary, reviewing the inherent characteristics of the children and their intervention program shows that a higher IQ and the development of communication and language skills at an earlier age are the most powerful factors contributing to a rapid recovery. Other factors, such as young age at identification and intervention, presence of mild to moderate symptoms, and the collaboration of families in the administration of education may have contributed to this favorable outcome. However, the lack of an appropriate control group limits our interpretation of these characteristics.

Another noteworthy point is the variability in the length of time from diagnosis to optimal outcome. The minimum time from intake to the loss of diagnosis was 6 months, while the maximum was 8 years. This indicates that OO is possible even in middle childhood. It is necessary to follow up with the individuals with ASD to determine if additional individuals lose the diagnosis as well as determining what happens to the children who move off the spectrum.

This study has some limitations, such as the lack of a diagnostic assessment with a structured interview form, such as the Autism Diagnostic Interview-Revised (ADI-R) [43] and the Autism Diagnostic Observation Schedule (ADOS) [44], due to the unavailability of these instruments in Turkish. Another limiting factor is that the information regarding the time and quality of the home-administered program was reported by the families and was not under our direct observation. The third factor that affects the generalizability of these results is the sample-specific characteristics of our group. This group is well educated, and the majority is from the upper-middle class, which has access to high quality health services. Additionally, the children had very high IQ scores, which again limits the generalizability of our results. Another limitation is that because this group was in a regular clinical follow-up, it was not possible to assess the children at uniform time points in terms of developmental periods, making it difficult to determine whether there were specific developmental factors that accounted for optimal outcomes at different time points. Finally, the lack of a control group limits our interpretation of the characteristics of children who can achieve an optimal outcome.

Nevertheless, this study has some strengths. One of the most important points is that it includes children who were followed by our study group; thus, the information given here is clinically based and prospective. All diagnostic and follow-up processes were completed by taking information from different and independent informants and by observing the child. Additionally, this study provides a follow-up intervention model for countries and settings with relatively limited resources for the treatment of ASD. Moreover, it provides information from a unique setting and culture, thus drawing attention to the need for new models of intervention to help families in low- and middle-income countries.

When interpreting the results of this study, it should be noted that it is not an effectiveness study; therefore, it would be difficult to predict the number of children who may benefit from the program. We have many children who are in similar programs (both ABA and naturalistic behavioral programs) and who remain in the ASD spectrum; we do not claim that this program represents a new gold standard in departing from the autism spectrum. Even so, we elucidated two main points; first, with early identification and an appropriate intervention program, an optimal outcome is possible for some children with ASD and, finally, in settings where there is a lack of community service for this group, motivating, teaching, and close collaboration with all available family members and schools may help to improve the clinical characteristics of this group and potentially even discard autism.

## 5. Conclusion

This study documented the data of children who lost a diagnosis of autism. It could be concluded that high IQ and the development of language at an early age are the most powerful factors contributing to optimal outcomes. Additionally, early identification and intervention, relatively mild symptoms, and, finally, a high motivation of parents in the administration of home programs are other common characteristics of this group.

It is important to mention that, in settings where there is a lack of resources for intervention programs, motivating and closely supervising family members could help to improve the social communicative abilities of this group.

## Figures and Tables

**Table 1:** Education program of the patients.

	Values
Years attending special education	2.00 ± 1.54 years
Psychiatric follow-up	3.10 ± 2.08 years
Special education hours per week	1.94 ± 1.61 hours
Number of home program providers	2.14 ± 1.41 persons
Number of patients attending mainstream education/nursery	31 patients (79.48%)
Age at starting mainstream education	3.00 ± 1.46 years
Number of patients attending speech therapy	15 patients (38.46%)

**Table 2:** Changes in CARS items.

CARS item	Initial score mean	Initial score standard deviation	Final score mean	Final score standard deviation	*P* value
(1) Relating to people	2.40	0.37	1.14	0.23	<0.0001
(2) Imitation	2.45	0.46	1.06	0.20	<0.0001
(3) Emotional response	2.19	0.39	1.10	0.20	<0.0001
(4) Body use	2.26	0.54	1.06	0.17	<0.0001
(5) Object use	2.46	0.52	1.29	0.42	<0.0001
(6) Adaptation to change	2.03	0.54	1.27	0.36	<0.0001
(7) Visual response	2.27	0.44	1.14	0.23	<0.0001
(8) Listening response	2.41	0.32	1.18	0.27	<0.0001
(9) Taste, smell, and touch response	1.63	0.48	1.14	0.28	<0.0001
(10) Fear or nervousness	1.49	0.41	1.30	0.39	0.03*
(11) Verbal communication	2.69	0.42	1.25	0.30	<0.0001
(12) Nonverbal communication	2.41	0.36	1.14	0.23	<0.0001
(13) Activity level	1.73	0.59	1.91	0.79	0.13**
(14) Intellectual response	2.00	0.53	1.12	0.21	<0.0001
(15) General impressions	2.41	0.38	1.09	0.19	<0.0001

*Not significant after Bonferroni correction.

**Not significant.

**Table 3:** Results of the outcome measures.

	Values
Mean age at optimal outcome	5.11 ± 1.95 years
Time to optimal outcome (range)	2.71 ± 1.67 years (0.5–8 years)
Optimal outcome <4	9 patients (23.07%)
Optimal outcome ≥4	30 patients (76.9%)
Mean CARS score	18.01 ± 1.76
Mean ABC score	7.53 ± 7.83
Mean IQ score	116.70 ± 18.88

**Table 4:** Correlations of variables with length of time to optimal outcome.

	Time to recovery	
	Pearson correlation coefficient	*P* value
Final IQ	−0.491	0.009
Number of weekly special education sessions	0.173	NS
Age at diagnosis	0.179	NS
Use of first words (age)	0.607	0.027
Use of first phrases (age)	0.734	0.006
Initial CARS scores	0.274	NS
